# Participatory approaches involving community and healthcare providers in family planning/contraceptive information and service provision: a scoping review

**DOI:** 10.1186/s12978-016-0198-9

**Published:** 2016-07-22

**Authors:** Petrus S. Steyn, Joanna Paula Cordero, Peter Gichangi, Jennifer A. Smit, Theresa Nkole, James Kiarie, Marleen Temmerman

**Affiliations:** Department of Reproductive Health and Research, World Health Organization, Geneva, Switzerland; International Centre for Reproductive Health, University of Nairobi and Ghent, Nairobi, Kenya; Maternal, Adolescent and Child Health Research Unit, Durban, South Africa; University Teaching Hospital, Lusaka, Zambia

**Keywords:** Community participation, Healthcare provider, Unmet need, Family planning, Contraception, Review

## Abstract

As efforts to address unmet need for family planning and contraception (FP/C) accelerate, voluntary use, informed choice and quality must remain at the fore. Active involvement of affected populations has been recognized as one of the key principles in ensuring human rights in the provision of FP/C and in improving quality of care. However, community participation continues to be inadequately addressed in large-scale FP/C programmes. Community and healthcare providers’ unequal relationship can be a barrier to successful participation. This scoping review identifies participatory approaches involving both community and healthcare providers for FP/C services and analyzes relevant evidence. The detailed analysis of 25 articles provided information on 28 specific programmes and identified three types of approaches for community and healthcare provider participation in FP/C programmes. The three approaches were: (i) establishment of new groups either health committees to link the health service providers and users or implementation teams to conduct specific activities to improve or extend available health services, (ii) identification of and collaboration with existing community structures to optimise use of health services and (iii) operationalization of tools to facilitate community and healthcare provider collaboration for quality improvement. Integration of community and healthcare provider participation in FP/C provision were conducted through FP/C-only programmes, FP/C-focused programmes and/or as part of a health service package. The rationales behind the interventions varied and may be multiple. Examples include researcher-, NGO- or health service-initiated programmes with clear objectives of improving FP/C service provision or increasing demand for services; facilitating the involvement of community members or service users and, in some cases, may combine socio-economic development and increasing self-reliance or control over sexual and reproductive health. Although a number of studies reported increase in FP/C knowledge and uptake, the lack of robust monitoring and evaluation mechanisms and quantitative and comparable data resulted in difficulties in generating clear recommendations. It is imperative that programmes are systematically designed, evaluated and reported.

## Background 

### Introduction

Unmet need for contraception remains high and is highest among the most vulnerable with about 225 million women and girls having an unmet need for modern contraception [1]. Additionally, many women using contraceptives are not satisfied with their method, potentially putting them at risk for discontinuation of a contraceptive method. The International Conference on Population and Development (ICPD) emphasized that the commitment to human rights (HR) in the delivery of sexual and reproductive health services should not be compromised to reach quantitative goals [2]. Voluntary use, informed choice and quality must remain at the fore as efforts accelerate to increase contraceptive uptake.

Community participation has been recognized as a key component in defining essential healthcare since the Alma-Ata declaration [3]. The World Health Organization’s (WHO) more recent recommendations for “Ensuring human rights in the provision of contraceptive information and services” [4] included participation as one of the nine key principles identified. Participation has been recognised as a precondition for sustainable development and for ensuring good-quality care and increased use of services [4, 5].

Participation is defined as the active involvement of affected populations in decision-making, implementation, management and evaluation of policies, programmes and services [5]. Although participatory approaches have been implemented in health programmes, participation has remained inadequately addressed in large-scale family planning/contraceptive (FP/C) programmes [6].

Previous experiences in community participation in health demonstrated that community and healthcare providers’ (HCP) unequal or conflictual relationship may act as a barrier to successful community participation, i.e., unaligned priorities and the inability of community members to communicate their needs and health professionals not being receptive [7]. Ensuring engagement from both the community and health providers in the design, implementation and evaluation may increase programme efficacy and sustainability. It may empower healthcare providers to implement realistic changes that reflect the needs of the community [8–10].

This scoping review was undertaken to identify participatory approaches involving both community and healthcare providers for FP/C services and to synthesize and analyse relevant evidence.

## Methodology

A scoping review methodology, which aims “to map rapidly the key concepts underpinning a research area and the main sources and types of evidence available” was used [11]. The writing group developed a review protocol, formulated the PICO questions and synthesized the findings through an iterative process.

Five online databases; PubMed/Medline, Cochrane Central Register for Controlled Trials, Global Index Medicus, Popline and EBSCO were searched up to 30 April 2015. A targeted Google search of websites of international, governmental and non-governmental organizations, funding bodies, research groups active in reproductive health, specifically FP/C was also conducted (See Appendix). Specific focus was directed to institutions doing research in or implementing participatory health programmes.

### Inclusion criteria

**Fig. 1:**
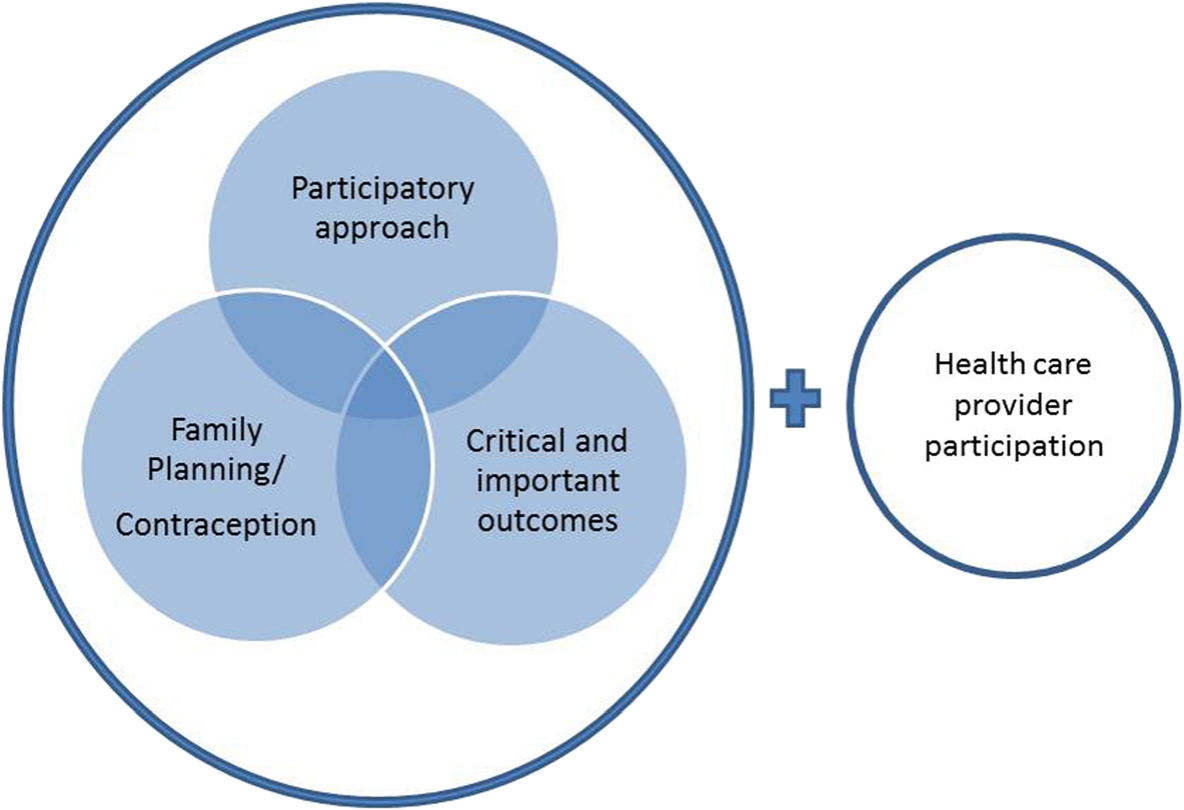
Key concepts used to develop the search strategy

The following key concepts were used to develop a search strategy: “Family planning/contraception”, “community participation approaches” and critical and important programme outcomes (Fig. 1).

For the search strategy, the authors did not initially include the healthcare provider component to ensure articles that did not explicitly claim to address community and health provider collaboration but, nevertheless, use mechanisms involving the two groups. Community was defined in the broadest way and included women, girls, men, individuals or groups representing their needs, using or needing FP/C services.

The writing group identified and graded possible outcomes. The critical outcomes included unmet or met needs, utilization and uptake of FP/C, satisfaction with method and services and health outcomes. Other outcomes considered include the impact on human rights principles, on empowerment of community members in managing their own reproductive health and on social determinants of health.

Studies included in the review were not restricted by country of origin, date or language (where access to translation fitted within the review timeframe). Published and grey literature on relevant studies and programmes of all designs were included. Secondary analyses were considered to gather general information and include programmes conducted in the 1960s to the early 1980s, which were rarely documented.

### Exclusion criteria

Programmes that did not explicitly address the relationship between community or service users and providers were excluded from the review, such as articles where community participation is defined solely as a mechanism for extending the health system. These include community-based distribution of services through health volunteers who do not influence the design or priorities of the programme.

Studies focusing on participation in other health services such as maternal and child health and sexually transmitted infection (STI), including HIV prevention programmes that are not integrated with FP/C, were not included.

Studies using qualitative research methodologies to gather user perspectives on FP/C issues were excluded, unless they were conducted as part of a community-informed intervention.

## Results

**Fig. 2:**
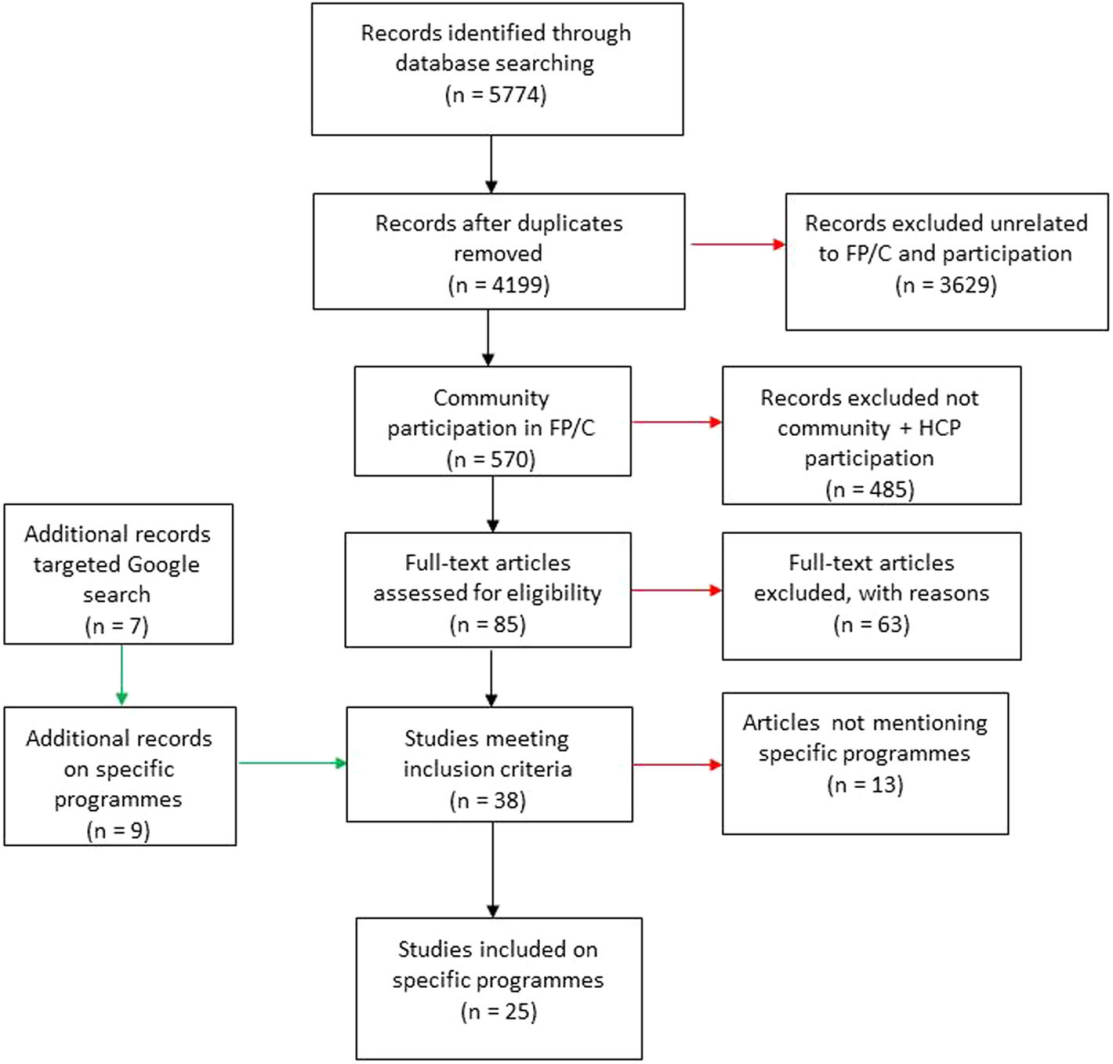
Flow diagram of article inclusion and exclusion

5774 articles were identified through the database search (Fig. 2). Following elimination of duplicates and irrelevant articles, 85 articles were retrieved for full article review of which 63 articles did not meet the inclusion criteria. Seven additional publications were identified from the targeted Google search. The programmes identified in the results were also searched resulting in inclusion of nine other sources.

Thirty-eight articles met the inclusion criteria, of which 25 articles published between 1972 and 2014, reported on 28 specific programmes [12–36] (See Table 1). 13 publications, which did not mention specific programmes, were also consulted for background information [7, 9, 10, 37–46].

**Table 1:** List programmes by approach showing three main types

Approach:	Programme name - Country	Reference(s)	Study Design	Programme initiation	Participants/Members	Services provided
Health committee: Advisory committee	Emory University Family Planning Program - United States	Bradshaw 1972 [17]	Case study	Government initiated through federal guidelines, welcomed by professionals.	FP patients and community representatives with guidance and assistance from health professionals (when requested by committee members)	• Motivation for FP • Advisory role in FP programme
Health committee: Supervisory committee	Tenewek Hospital Community Health Programme - Kenya	Jacobson 1989 [25]	Case study	Initiated by researchers, initial contact with community was made through congregations affiliated with the same mission as the hospital. Church leaders in villages interested in the project were involved.	Church leaders, village leaders and members, community health worker, community health programme staff	• Community-based health and FP services • Training of providers
Health committee: Supervisory committee	Bouddha-Bahunepati Family Welfare Project - Nepal	Askew 1989 [12]	Secondary analysis based on case studies	Initiated by British expatriate nurse who established links with local FPA.	Committee membership restricted to certain users (volunteers), district level FPA staff responsible for project implementation	• Motivation for and delivery of FP • MCH and basic health services • Integration and drinking water • Afforestation • Horticulture • Farming
Health committee: Supervisory committee	Community-managed Rural Family Project - Sri Lanka	Askew 1989 [12]	Secondary analysis based on case studies	Initiated by the FPA to expand pilot study	Local community leaders supervising health volunteers. District level staff responsible for implementation.	• Motivation for FP • Organization of committees • Community infrastructure
Health committee: Co-management	Special Project with the Bangladesh Agricultural University, Mymesingh - Nepal	Askew 1989 [12]	Secondary analysis based on case studies	Initiated by a university medical officer who established links with the local FPA	Committee membership restricted to certain service users. Community based project officer responsible for project implementation.	• Motivation and delivery of FP • MCH and basic health services • Revolving loan fund • Nonformal education
Health committee: Co-management	Kundam Family Welfare Project - India	Askew 1989 [12]	Secondary analysis based on case studies	Initiated by FPA India to replicate a pilot project in Karnataka	Committees with different types of members, including youth and women. Community based project coordinator manages implementation.	• Motivation for and delivery of FP • Health education • Nonformal education • Revolving loan fund • Skills training • Community infrastructure
Health committee: Co-management	Community-Managed Welfare Projects Kaligonj - Bangladesh	Askew 1989 [12]	Secondary analysis based on case studies	Initiated by FPA Bangladesh based on guidelines by IPPF.	Village action committee comprising of health volunteers. Representatives from village participate in management committee. Project officer based in community responsible for project implementation.	• Motivation and delivery of FP • Irrigation and drinking water • Sanitation • Pisiculture • Horticulture • Poultry and livestock • Revolving loan fund • Functional literacy
Health committee: Co-management	Community-Managed Welfare Projects, Deokhuri - Nepal	Askew 1989 [12]	Secondary analysis based on case studies	Initiated by FPA Nepal based on guidelines by IPPF.	Village action committee comprising of health volunteers. Representatives from village participate in management committee. District level FPA staff responsible for project implementation.	• Motivation and delivery of FP • Irrigation and drinking water • Sanitation • Pisiculture • Horticulture • Poultry and livestock • Revolving loan fund • Functional literacy
Health committee: Co-management	Community Health Department of the Chogoria Hospital - Kenya	DeBoer 1989 [20]	Case study	Initiated by hospital management	Community leaders from different altitude zones, CHD	• Motivation for and delivery of FP (initial focus) • Health education • Education and information programme for men and grandparents • MCH services • curative services
Health committee: Co-management	Bamako Initiative - Senegal	WHO 1999 [36], Wickstrom 2006 [35]	Case study	Jointly developed by WHO and UNICEF was adopted and implemented by governments in the African Region, including the Senegal example		• Community co-financing and co-management of the provision low-cost essential drugs and supplies • Priority areas include: HIV/AIDS, tuberculosis, malaria, maternal health and malnutrition.
Health committee: Co-management	Santa Barbara Project	Diaz 1999 [21]	Case study	Initiated by local municipality in Brazil in collaboration with the NGO, Centro de Pesquisas em Saúde Reprodutiva de Campinas (CEMICAMP)	Municipal health secretary, service providers, CEMICAMP representatives, members of the women’s group SOS Mulher (women's group created for the project)	• Training • Restructuring of provider roles and service delivery patterns, • Creation of referral centre • Delivery of FP - introduction of injectable contraceptive and vasectomy services and management process
Health committee: Co-management	Swarnirwar Program	Islam 2001 [24]	Case study	Supported by Pathfinder International	Swanirwar officials, FP committees, Project officer and volunteers, Government FP workers, the family planning inspector (FPI) and local elite attend meetings	• home-based FP service • operate satellite clinics • Immunization
Health committee: Co-management	Family Planning Facilitation Program	Islam 2001 [24]	Case study	Requested by the government, project initiated by the Health and Population Division of the NGO, Bangladesh Rural Advancement Committee (BRAC)	Community leaders	• Motivation and demand creation for FP • FP service provision
Health committee: Co-management	Health and Population Sector Programme	Sarker 2001 [31], Normand 2002 [27],	Programme evaluation	Initiated by the government through the Ministry of Health and Family Welfare.	A community group for each community clinic was established with individuals in that catchment area; service providers (Health Assistant or Family Welfare Assistant)	• Essential Services Package (ESP): RH, Child Health Care, Communicable disease control, limited curative care, and Behaviour Change Communications
Health committee: Co-management	Foundation of Research in Health system Project - India	FRHS, 2004 [22]	Experimental design	Initiated by the NGO, Foundation of Research in Health system with government encouragement.	Community members suggested by health providers and approved by adults in the community participated as committee members, health staff, NGOs, community facilitators	• Raise awareness about health issues related to reproductive and child health service • Promote new services • Identification of community needs
Health committee: Co-management	Navrongo Study - Ghana	Solo 2005 [33] Solo 2005 [34],	Quasi-experimental study	Initiated by the Ministry of Health. Conducted by the Navrongo Health Research Centre	Council of chiefs and elders – traditional male leaders	• FP and reproductive health • Outreach services, including education, referrals and limited range of health services
	Nkwanta Initiative - Ghana	Awoonor 2004 [13] Solo 2005[33], Solo 2005 [34]	Quasi-experimental study	Ten Ministry of Health regional directors were informed of progress in Navrongo and district health management team were invited to observe the Navrongo Project firsthand.	Male community leaders identified among elected officials, teachers and clerics	• FP and reproductive health • Outreach services, including education, referrals and limited range of health services
	Community Health Planning and Services - Ghana	Solo 2005* [33], Solo 2005* [34], Baatiema 2013* [14], MOH Ghana 2009** [26]	*Case study, **programme evaluation	Ministry of Health through the Ghana Health Service, nation-wide implementation based on findings of the Navrongo Study and Nkwanta Initiative.	Community health nurse acts as community health officer and works with community members of the committee and volunteers.	• promotion and prevention, management of common ailments and their referrals and, case detection mobilization and referral • curative services, for instance malaria, HIV/AIDS • key MCH services, including, growth monitoring, ANC and FP services
Health committee: Community managed	Family Welfare Centre Project - Pakistan	Askew1989 [12]	Secondary analysis based on case studies	Initiated by local FPA	Community leaders who are self-selected and district level staff responsible for implementation	• Motivation for and delivery of FP • Training traditional birth attendants • Social education for women • MCH services • Adult literacy • Skills training
Implementation Team: Quality Improvement Team	Better Life for Youth - Nepal	Save the Children 2004 [32]	Project Report	Initiated by research project team from NGO, Save the Children in collaboration with BP Memorial Health Foundation and Nepal Red Cross Society.	In- and out-of school youths from 10 to 21, health providers, research team	• IEC, peer education, training • FP/C promotion • Prevention of premarital pregnancy and early pregnancy • STI and HIV prevention • Telephone hotline counselling
Implementation Team: Family Planning Implementation Team	Uganda Child Spacing Program	Patterson 2008 [28]	Project Report	Project initiated by Minnesota International Health Volunteers (MIHV)	District Health Officer/representatives, Community Development Officer, representative from Ministry of Gender, Labor and Social Development, MIHV staff who function as Program coordinator (nurse/midwife) and Program officer (role - community health educator), representative of FP-CHW selected by their peers and MIHV staff.	FP promotion and service provision
Self-help organization collaboration	Maternal Child Health-Family Planning program - Bangladesh	Bhuiya 1998 [15]	Experimental Study	Initiated by International Centre for Diarrhoeal Disease Research	Indigenous village-based self-help organizations (SHO), health service providers, research group	FP and maternal and child health services
COPE: Quality Measurement Tool	Reproductive health services' quality improvement programme -Tanzania -	Bradley 2002 [16]	Case study	Project initiated by Reproductive Health care programme consortium that include the Ministry of Health, UMATI and EngenderHealth	Health supervisors, health providers and community (as sources of information, except in sites where community representatives have been invited to be more involved)	Reproductive health services
COPE and JHU Bridging approach	Pont d'Or Project (Senegal Maternal/Family Planning Project) - Senegal	Pollock et al. 2003 [29] and Wickstrom 2006 [35]	Programme evaluation (mid-term)	Pilot program created from the SM/PF Project initiated by Project team, Management Sciences for Health.	Providers and clients, research group, government, each level of health management is involved.	FP and maternal health services
Community score card	Tanzania community score care	CARE 2012 [19]	Programme report	Approach developed and implemented by international NGO, CARE	Community representatives, healthcare providers	FP and maternal health services
Client-friendly FPA	Family Planning Associations – St Lucia, Guyana, Belize (three examples)	Campbell 1998 [18]	Project report	Initiated by International Planned Parenthood Federation Family Planning Associations in each country.	FPA staff with community members	FP services
Participatory management	The Greater Soweto Maternal Child Project - South Africa	Ramontja 1998 [30]	Project report	Initiated by International Centre for Diarrhoeal Disease Research	Community health workers, Local Soweto Health Authority, Civic Association and communities served	• HIV/AIDS counselling • Advice on family planning
Social Network Package	Tékponon Jikuagou - Benin	IRH 2014 [23]	Quasi- experimental Study	Research project conducted by Institute for Reproductive Health Georgetown University.	Community groups, individuals, FP providers, members of research group	Motivation for FP

The reviewers developed a data charting form to determine which variables to extract [47] from the 13 studies which were conducted in Asia, predominantly South Asia, ten in Africa and five in North and South America.

### Approaches

Approaches for community and HCP participation in FP/C programmes from the review can be categorised into three types. The first consists of establishing a group of individuals who link the community and health service (health committees) or conduct specific actions to achieve pre-defined goals (implementation teams).

The establishment of health committees was the most common approach (Table 1). In 11 of the 17 health committee examples, community members co-managed certain or all project activities [12–19, 35]. In other examples, health committees supervised activities such as selection and management of community health volunteers [25].

Other health committees consist of community members and representatives providing recommendations [32] or making up a managerial committee working with health providers who implement activities [12, 17].

Two examples of implementation teams focused on purposefully engaging inter-sectoral collaborative teams. They were brought together to identify clear action plans and conduct or facilitate implementation [28, 32].

The second type of participatory approach involved identifying existing community structures to optimise use of health services (Table 1). In the Maternal Child Health – Family Planning programme, a participatory needs assessment was conducted and the plan of action created and implemented in collaboration with existing community-based self-help organizations [15].

The third type of approach involved operationalization of tools or frameworks developed by researchers or NGOs to facilitate community and healthcare provider collaboration for quality improvement, accountability or governance (Table 1). These tools, as shown in three examples below, propose different means of overcoming or leveraging the complex relations that exist between and amongst community members and HCP.

The Community-oriented, Provider efficient (COPE) framework is based on 10 key elements, which include on the one hand, clients’ rights to information, access, choice, safe services, privacy and confidentiality, dignity opinion and comfort, continuity of services, and on the other, staffs’ need for good management, good supplies and infrastructure, information and training [29, 35]. A participatory governance approach using community scorecards is another example [19]. Here, community members and HCP separately score indicators of coverage, quality and equity of FP/C services. Both groups are then given an opportunity to discuss and identify solutions through interface meetings. Once an action plan has been identified, both agree on the roles, set timelines and develop a monitoring and evaluation plan. Another project tested a social network package to raise awareness about FP/C and improve access to services, based on the recognition that women and men are members of formal and informal social networks who influence their reproductive health choices [23].

### Participation in FP/C programmes

Integration of community and HCP participation in FP/C provision was implemented in three ways. The first consisted of integrating participation in FP/C-only programmes [12, 16, 18, 23, 24, 28]. The second used programmes that are FP/C-focused but also provide other services [12, 24, 27, 31] and, finally, through fully integrated programmes [12, 15, 19, 20, 22, 29, 32, 35] where a range activities, including FP/C were provided as part of a service package.

Early participatory programmes that introduced FP/C in settings where services were minimal or non-existent led to limited success [20]. A programme evaluation showed that focus on FP/C provoked the mistrust of community members and following a restructuring, an integrated service that included maternal and child health services was put in place [20]. Integration with other health and/or developmental activities facilitated FP/C service and information delivery. Events focusing on children’s health, where both men and women were more likely to attend, assisted in ensuring effective outreach [28]. More recent FP/C-only services are showing promising results with improvement in quality of care (QoC), accountability and governance [16, 23, 28].

### Monitoring and evaluation

Measurement and reporting of outcomes were inconsistent making the data gathered difficult to put on a quantitative scale. For most, a combination of quantitative and qualitative methodologies was used [20–22, 25, 27, 28, 31, 32, 34]. Comparisons between baseline and end line surveys, as well as, regular programme reporting were used to gather data. Interviews and focus group discussions were conducted. Two programmes embedded monitoring and evaluation strategies within the intervention design, such as the community scorecard or COPE [19, 29, 35]. A framework for measuring impact, spidergrams, was tested in one site of the Community-based Health Planning and Services (CHPS) implemented in Ghana [14]. Several studies provided limited or no information on monitoring and evaluation [12, 17, 18, 24].

### Outcomes

Measurement and reporting of outcomes were inconsistent making the data gathered difficult to put on a quantitative scale. Out of the 28 programmes, 11 did not have information on monitoring and evaluation.

Early attempts of health committees lacked strong evidence-based foundations and as a result the operationalization of community participation—from recruitment, role to implementation of activities and the ways in which they link to the health system—were conducted randomly [12, 21]. Analysis of these programmes showed mixed results and, at times, failure to initiate meaningful and sustainable community participation. In the Emory FP programme, the recruitment of participants that included identification of stakeholders and the process of engaging them, was done randomly [17]. Due to unequal experiences and knowledge among the members, participation was uneven [12, 17]. More recent examples of committees have built on the lessons learned of past experiences and have made provisions to address these issues [32].

Decreasing unmet need was not specifically addressed by any of the programmes or projects included with the exception of one recent study but outcomes were not published yet at the time of this review [23].

Increase in FP/C knowledge, utilization and uptake was reported in 11 of the programmes [12, 15, 19, 22, 28, 29, 33]. For the majority, a clear causal relationship between the outcome and participatory component could not be identified and only four reported quantitative data.

### Community and healthcare provider dynamics

Although not one of the outcomes pre-defined for the review, the majority of the programmes reported on outcomes related to the participatory mechanism itself and client-provider relations [12, 17–19, 22, 29, 35, 36]. The reported impact on community and healthcare provider participation was mixed. An analysis of seven case studies, found that overall the degree of community involvement in designing the projects had been limited [12]. Healthcare providers working closely with the community had the greatest influence in the decisions made. In these examples, the health committees provided support and helped legitimize the action plans. In other examples, improvement in community and healthcare provider relation remained limited. Community members reported that they felt treated paternalistically by staff and professionals were frustrated by the recommendations made by committee members that lacked focus or specific strategies for implementation.

Nine of the programmes resulted in positive outcomes on the participation between community and healthcare providers itself [15, 16, 21, 22, 25, 28, 29, 32, 36]. In these cases, the interventions were successful in identifying needs of the community e.g., knowledge gaps to be addressed [17], use of both male and female community health workers to reach a wider range of users and potential users [28] and engaging key influencers who play a significant role in women’s reproductive health choices such as their husbands or their mother-in-laws [20]. They were also effective in implementing solutions identified by them or jointly with healthcare providers and with their participation, e.g., suggesting Family Planning Days to improve outreach to youth and determining the acceptable limits of FP-community health workers (CHW) [28] and participatory action planning and management of services to improve quality of services and rural and regional radio programming as a means of educating wider public on maternal health and family planning [29, 35].

Increasing the link between health providers and the community that they serve led to greater awareness from both sides about the issues, barriers and the needs leading to the identification of appropriate actions and solutions [28]. Interventions helped bridge the gap between providers and their clients in the Better Life for Youth [32] and Bamako initiative [19, 36] examples. The Pont d’Or project led, not only to the identification of barriers, but also to finding and implementing solutions [29, 35]. These practical outcomes were accomplished through COPE, which was an important relationship building exercise. COPE was also reported to be successful in promoting new levels of understanding of QoC [16].

One study showed initial promise in involving women. The SOS Mulher women’s group was formed very quickly following the start of the Santa Barbara project, but it was short-lived [35]. This was due to the difference in socio-economic status among the women in the group and the users of the service, as well as, external factors [21]. In this case, the municipal election discouraged participation for fear of being seen as campaigning for the official in place [21].

### Youth participation

Youth participation was addressed in three studies [12, 22, 32]. One example saw young people participating in committees for FP and development activities, which resulted in the community recognizing them for their active involvement and the benefits they brought [12]. No further information was given on the specific role and extent of responsibilities given to the young people [12]. An effective youth-adult collaboration could be seen in Quality Improvement Teams where youth members and HCP worked together in a Youth-Defined Quality process that involved collaborating on problem identification, information, education and communications (IEC) and training activities and programme evaluation [32]. Foundation for Research in Health Systems (FRHS) reported engaging youth representatives within the village-level health committees to ensure inclusion of youth needs in their assessment activity [22].

### Sustainability

A quasi-experimental study, the Navrongo Study, conducted in 1993 to test effective mechanisms for health service delivery is one of several examples showing that scaling-up research projects is possible [33, 34]. The programme was tested for replicability in a non-study site [13]. Following the successful implementation, the CHPS programme as developed and implemented in all 10 regions and 110 districts of Ghana [35]. Lessons learned from the successful scaling up, include the importance of sharing outcomes and findings between regions. The programme in the non-study site started with the local district health management team visiting the study-site for training. Regional exchanges were being explored at the time of the report writing [26]. Additionally, the successful implementation of CHPS showed that the order of activities is important i.e., it should begin with community dialogue. Non-financial incentives, such as certification mechanisms, have been successfully used to avoid over-dependence on external funds. This creates a healthy competition between neighbouring communities encouraging community members and HCP activities [12, 29, 35]. FRHS’ project had formed 64 committees during the study period, of which 61 committees were still active with minimal involvement from community facilitator, a year after the project ended, showing their sustainability [22].

## Discussion

This review presents an overview of how community and HCP participation have been implemented. The review was conducted using an extensive search strategy and aimed to be as comprehensive as possible in gathering and analysing participatory approaches involving both community and healthcare provider. The quality of evidence gathered was mixed and in certain areas comparisons were impossible, making it difficult to make definitive conclusions. No conclusions could be made on whether integrating participatory interventions in FP/C is more effective in integrated, FP/C-only, or FP focused services. Five of the 11 FP-only programmes did not report outcomes. Five out of six FP/C focused examples were from the same secondary analysis [12]. However, the evidence seems to suggest that integrated services may be better suited when using community and HCP participation to introduce FP/C service in a setting where it has been non-existent. Introducing participation through integrated services rather than FP/C only may also be better-suited when community structures are still weak or missing. More recent attempts have yielded promising results in terms of quality improvement and governance in FP/C-only services. The difficulty in generating clear recommendations may be linked to the lack of quantitative and comparable data resulting from the lack of robust monitoring and evaluation mechanisms to measure empowerment outcomes and trace causal links between components of an intervention and the outcomes. This, in turn, may be closely linked to the lack of consensus on why participatory interventions are needed and implemented.

Analysis of studies on specific programmes and projects show that the rationales behind the interventions are varied and may be multiple. Most projects and programmes were initiated by researchers, NGOs or the health service who have clear objectives of improving FP/C service provision or increasing demand for services [12, 19–25, 28, 29, 32, 35]. Several programmes explicitly aimed to facilitate or maximise the involvement of community members or service users [22, 35, 36]. In some examples, improving socio-economic conditions and increasing self-reliance or control over sexual and reproductive health are combined [12, 18, 20, 24]. There were also initiatives aiming to promote an enabling environment for participation with a focus on improving community and provider relations and promoting capacity building, information and education [12, 32].

As shown in this review, implementation of community participation may not fall clearly within either one of the main typologies identified, which are participation as a means of improving health service delivery and participation as an empowerment process, wherein community members take more control of their own health [9, 10, 12, 44]. These two very different aims impact the way programmes are designed and what role community members take on.

This finding falls in line with the analysis proposed in some of the background articles. Analyzing organizational factors within participatory interventions in national FP/C programmes, Askew concluded that participation is predominantly seen as a means to an end because programmes are explicitly responding to specific demographic policies by creating demand for sustained use of FP by increasing the social acceptability of services [31]. Participation of community members in planning and implementation have not been strongly pursued because it is extremely complex and could even reduce efficiency given the bureaucratic structures of programmes, at least in the beginning [31].

Russell et al. [44] in presenting their framework for analysing community engagement in reproductive health and family planning defines the goal of participation as a collaborative partnership among community, NGOs and government in which community members serve as champions and advocates for quality programmes that take root and are sustained over time.

Other discussions on the rationale of participation have proposed a move away from defining participation as being exclusively within either one of these two main typologies. Askew in other publications posited that taken to their extremes, the two typologies may be undesirable and may not even be feasible [7, 12]. It was concluded that community participation should be seen as a partnership approach to service provision and not a means to create self-sufficiency in the community while reducing the obligations of the formal health sector [7]. Rifkin, proposed a functional definition of community participation [9]. She recommended that focus be given to questioning the concrete components of community participation, such as why participation, who participates and how people participate. Responding to these questions allow researchers and programme managers to make clear statement about programme objectives. Maclean also suggested a parallel path arguing for the creation of an approach with two complementary sets of objectives, one programme-focused and the other focused on building community capacity [10].

Based on the findings of this review, evaluation of participatory interventions should include indicators for measuring the impact on community and healthcare provider relations. Indicators should also be, at least partially, identified by the service users and providers. Key intermediate outcomes need to be identified to ensure that the causality between intervention activities and health outcomes can be established.

## Conclusion

There is a paucity of rigorously evaluated studies on community and healthcare provider participation in FP/C. However, recent studies provide evidence that it is feasible for community and healthcare providers to collaborate and dialogue on FP/C, and that such interventions have shown promise in quality improvement and increasing accountability.

The conceptualization and implementation of community participation is evolving. With the realization that communities are complex and that individuals exist within social structures, interventions may be better able to address the challenges identified in the past.

There is a need to continue evaluating participatory interventions and provide robust evidence to guide health ministers, programme managers, health providers and community members in addressing unmet need for FP/C. A better understanding of the relationship between outcomes and participation involving community and HCP is needed to help ensure that individuals’ sexual and reproductive health and rights remain in the fore as efforts to address unmet need for FP/C accelerate.

Further research, improved evaluation and critical examination of all components of participatory programmes are essential to improve understanding of community participation approaches in FP/C and their value in meeting unmet need for FP/C and to guide future programmes, scale-up and replication.

## Abbreviations

ANC, antenatal care; CHPS, community-based health planning and services; CHW, community health workers; COPE, client-oriented and provider efficient; FP/C, family planning and contraception; FPIT, family planning implementation team; FRHS, Foundation for Research in Health Systems; HCP, healthcare providers; HR, human rights; IEC, information, education and communication; MCH, maternal and child health; NGOs, non-governmental organizations; PAC, patient advisory committee; PICO, population, intervention, comparison, outcome; QI, quality improvement; QoC, quality of care; SMFP, senegal maternal and family planning; STI, sexually transmitted infections; WHO, World Health Organization

## Appendix 1: List of websites searched in targeted Google Search (accessed 13–16 April 2015)

• Acquire Project/EngenderHealth

• CARE

• DFID working with Scaling Up Family Planning in Zambia

• FHI360

• FP2020

• Guttmacher Institute

• International Planned Parenthood Federation

• PATHfinder

• Population Council

• Save the Children

• Society for Family Health (PSI)

• UNFPA

• USAID

• WHO - using Google and WHO’s Institutional Repository for Information Sharing (IRIS)

## References

[1] Singh S, Darroch JE, Ashford LS (2014). Adding It Up: the Costs and Benefits of Investing in Sexual and Reproductive Health 2014.

[2] United Nations (1994). Report of the International Conference on Population and Development.

[3] World Health Organization (1978). Alma Ata Declaration.

[4] World Health Organization (2014). Ensuring Human Rights in the Provision of Contraceptive Information and Services.

[5] Sachs JD (2005). Investing in Development: a Practical Plan to Achieve the UN Millennium Goals: a Report to the UN Director-General.

[6] Marston C, Renedo A (2013). Understanding and measuring the effects of patient and public involvement: an ethnographic study. Lancet.

[7] Askew I, Carballo M, Rifkin S, Saunders D. Policy Aspects of Community Participation in Maternal and Child Health and Family Planning Programmes. World Health Organization. 1989:56.

[8] Lawn JE, Rohde J, Rifkin S, Were M, Paul VK, Chopra M (2008). Alma-Ata 30 years on: revolutionary, relevant, and time to revitalise. Lancet.

[9] Rifkin SB (1990). Community Participation in Maternal and Child Health/Family Planning Programmes.

[10] Maclean A. Community involvement in youth reproductive health and HIV prevention: A review and analysis of the literature. Family Health International/YouthNet. 2006.

[11] Arksey H, O'Malley L (2005). Scoping studies: towards a methodological framework. Int J Soc Res Methodol.

[12] Askew I (1989). Organizing community participation in family planning projects in South Asia. Stud Fam Plann.

[13] Awoonor Williams JK, Feinglass ES, Tobey R, Vaughan Smith MN, Nyonator FK, Jones TC (2004). Bridging the Gap Between Evidence‐based Innovation and National Health‐sector Reform in Ghana. Stud Fam Plann.

[14] Baatiema L, Skovdal M, Rifkin S, Campbell C (2013). Assessing participation in a community-based health planning and services programme in Ghana. BMC Health Serv Res.

[15] Bhuiya A, Yasmin F, BegUm F, Rob U (1996). Community participation in health, family planning and development activities a review of international experiences.

[16] Bradley JE, Mayfield MV, Mehta MP (2002). Participatory evaluation of reproductive health care quality in developing countries. Soc Sci Med.

[17] Bradshaw BRB, Mapp CBP. Consumer participation in a family planning program. Am J Public Health. 1972;62:969–72. 10.2105/ajph.62.7.969 PMC1530409 5039501

[18] Campbell L. Communities learn to take charge of their sexual lives and relationships. Curationis. 1997.

[19] CARE International. Women’s Lives, Women’s Voices: Empowering women to ensure family planning coverage, quality and equity. CARE International. 2012:1–24.

[20] DeBoer CN, McNeil M (1989). Hospital outreach community-based health care: The case of chogoria, Kenya. Soc Sci Med.

[21] Díaz M, Simmons R (1999). When is research participatory? Reflections on a reproductive health project in Brazil. J Womens Health.

[22] Foundation for Research in Health Systems (2004). Community Involvement in Reproductive Health: Findings From Research in Karnataka, India.

[23] Institute for Reproductive Health, Georgetown University (2014). Overcoming social barriers to family planning use: Harnessing community networks to address unmet need.

[24] Islam MA, Islam MM, Khan MA. Community Participation in Family Planning in Bangladesh: Prospects and Strategies. Journal of Health & Population in Developing Countries. 2001.

[25] Jacobson ML, Labbok MH, Parker RL (1989). A case study of the Tenwek hospital community health programme in Kenya. Soc Sci Med.

[26] Ministry of Health (2001). In-depth Review of the Community-based Health Planning Services (CHPS) Programme: A report of the Annual Health Sector Review 2009.

[27] Normand C, Iftekar MH, Rahman SA. Assessment of the community clinics: effects on service delivery, quality and utilization of services. Health Systems Development Programme. 2002.

[28] Patterson J (2008). Family planning implementation teams: building sustainable community ownership in rural Uganda.

[29] Pollock J, Bryant M, McKenney J, Sow A (2003). Senegal Maternal Health/Family Planning Project: Mid-Term Evaluation Report, November 2003.

[30] Ramontja R, Wagstaff W, Khomo N (1998). Urban community health workers: selection, training, practice and outcomes. Curationis.

[31] Sarker S, Islam Z, Hossain S, Saha NC, Routh S (2001). Operations Research on ESP Delivery and Community Clinics in Bangladesh.

[32] Save the Children (2005). Working to Improve the Reproductive and Sexual Health of Young People: Bhutan, Malawi, Nepal and Viet Nam.

[33] Solo J, Klitsch M (2005). Moving family planning programs forward: learning from success in Zambia Malawi and Ghana. The Repositioning Family Planning Case Study synthesis report.

[34] Solo J, Odonkor M, Pile J, Wickstrom J (2005). Ghana Case Study: “Give Them the Power”—A Repositioning Family Planning Case Study.

[35] Wickstrom J, Diagne A, Smith A (2006). Senegal Case Study: Promising Beginnings, Uneven Progress : a Repositioning Family Planning Case Study.

[36] World Health Organization. Review of the Implementation of the Bamako Initiative in Africa: Report of the Regional Director. World Health Organization; 1999:75.

[37] Japanese Organization for International Cooperation in Family Planning. Bringing women into the mainstream. Reproductive health and rights and women's empowerment are key words for JOICFP. Joicfp News. 1995; (254):1. 12290161

[38] Askew I, Khan AR. Community participation in national family planning programs: some organizational issues. Stud Fam Plann. 1990;21(3):127–42. 2375045

[39] Askew I, Lenton C (1987). Community participation in family planning: some suggestions for organisation development and management change.

[40] Askew I (1982). Assessment of local participation techniques in the provision of fertility regulating services.

[41] Askew I, Giridhar G, Ellen S, Kang JS (1989). The institutionalization of participatory projects”. Readings in population programme management.

[42] EngenderHealth/The AcquiredProject. Community Mobilization: Improving Reproductive Health Outcomes. 2006; 26;1–2.

[43] Lyons H. Planned parenthood as a concern of primary health care. Eschborn, Federal Republic of Germany, Deutsche Gesellschaft fur Technische Zusammenarbeit; 1983. p.173–82.

[44] Russell N, Igras S, Johri N, Kuoh H, Pavin M (2008). The Active Community Engagement Continuum.

[45] UN ESCAP (1988). Report of the study on the organizational issues in community participation in national family planning programmes: a comparative analysis of five countries in the ESCAP region.

[46] University of Exeter (1985). Research into community participation in family planning projects summary of progress 1981–1985.

[47] Levac D, Colquhoun H, O’Brien KK (2010). Scoping studies: advancing the methodology. Implement Sci.

